# Emergence of lineage ST150 and linezolid resistance in *Enterococcus faecalis*: a molecular epidemiology study of UTIs in Tehran, Iran

**DOI:** 10.3389/fmicb.2024.1464691

**Published:** 2024-10-14

**Authors:** Maryam Seyedolmohadesin, Mobina Kouhzad, Friedrich Götz, Maedeh Ashkani, Soheila Aminzadeh, Narjess Bostanghadiri

**Affiliations:** ^1^Department of Genetics, Faculty of Advanced Science and Technology, Tehran Medical Sciences, Azad University, Tehran, Iran; ^2^Department of Molecular Biology and Genetics, Izmir Institute of Technology, Izmir, Türkiye; ^3^Department of Microbial Genetics, Interfaculty Institute of Microbiology and Infection Medicine Tübingen (IMIT), University of Tübingen, Tübingen, Germany; ^4^Department of Biology, Central Tehran Branch, Islamic Azad University, Tehran, Iran; ^5^Toxicology Research Center, Medical Basic Sciences Research Institute, Ahvaz Jundishapur University of Medical Sciences, Ahvaz, Iran; ^6^Student Research Committee, Ahvaz Jundishapur University of Medical Sciences, Ahvaz, Iran; ^7^Department of Microbiology, School of Medicine, Iran University of Medical Sciences, Tehran, Iran

**Keywords:** *Enterococcus faecalis*, MLST, antibiotic resistance, biofilm, urinary tract infections

## Abstract

**Background:**

Urinary tract infections (UTIs) represent one of the most prevalent bacterial infections, with Enterococcus species now recognized as the second leading cause of these infections. This study focused on symptomatic UTI cases to investigate the risk factors associated with *Enterococcus faecalis* clinical isolates in patients from Tehran, Iran.

**Methods:**

Urine samples were collected from patients presenting with symptomatic UTIs. The identification of *E. faecalis* isolates was performed using standard microbiological techniques, with confirmation via polymerase chain reaction (PCR). Antibiotic susceptibility testing was conducted using the Kirby–Bauer disc diffusion method. The presence of virulence genes was determined through PCR, and biofilm formation was assessed using the microtiter plate method. Additionally, multi-locus sequence typing (MLST) was utilized to genotype linezolid-resistant isolates.

**Results:**

Out of 300 UTI cases*, E. faecalis* was identified as the causative agent in 160 instances. Notably, a high proportion of these isolates exhibited resistance to tetracycline (83.8%) and minocycline (82.5%). Linezolid resistance was observed in 1.3% (*n* = 2) of the isolates. Conversely, the highest susceptibility rates were observed for vancomycin, penicillin G, ampicillin, and nitrofurantoin, each demonstrating a 98.8% susceptibility rate. Biofilm formation was detected in 25% of the *E. faecalis* isolates. A significant majority (93.8%) of the isolates harbored the *efbA* and *ace* genes, with varying frequencies of *esp* (72.5%), *asa1* (61.2%), *cylA* (52.5%), and *gelE* (88.8%) genes. MLST analysis demonstrated that both linezolid-resistant isolates, characterized by strong biofilm formation and the presence of virulence genes, were assigned to the ST150 lineage, which has not been previously documented in clinical settings.

**Conclusion:**

The emergence of the ST150 clonal lineage, underscores its clinical significance, particularly in relation to linezolid resistance in *E. faecalis*. This study adds to the growing body of evidence linking specific clonal lineages with antibiotic resistance, highlighting the critical need for ongoing surveillance and molecular characterization of resistant pathogens.

## 1 Introduction

Urinary tract infections (UTIs) rank among the most prevalent infections acquired both in healthcare settings and within communities, with significant implications for patient health (Shahbazi et al., 2018; Shahkolahi et al., 2022). In 2019, more than 404.6 million individuals worldwide were diagnosed with a UTI, contributing to over 200,000 deaths globally (Codelia-Anjum et al., 2023; Shivaee and Mirshekar, 2019). UTIs are particularly common in vulnerable populations, such as pregnant women, the elderly, and sexually active individuals, who are prone to both community-acquired and healthcare-associated UTIs (CAUTIs and HAIs) (Shivaee and Mirshekar, 2019; Govindarajan and Kandaswamy, 2022). While *Escherichia coli* strains account for 80 to 90% of UTI cases, the rising incidence of *Enterococcus faecalis* strains in up to 20% of cases has garnered considerable attention (Taati Moghadam et al., 2021; Abdullah et al., 2023; Shahbazi et al., 2023). In Tehran, Iran, recent studies underscore the growing concern regarding UTIs. Research conducted at local hospitals has highlighted the high prevalence of *E. faecalis* among UTI patients, with significant resistance to commonly used antibiotics (Minaeian et al., 2020; Dadashi et al., 2021; Samani et al., 2021; Ma et al., 2021).

The emergence of *E. faecalis* as a prominent UTI pathogen is alarming, particularly due to its intrinsic resistance to a broad range of antibiotics, including aminoglycosides, cephalosporins, trimethoprim-sulfamethoxazole, and macrolides (Wojnicz et al., 2016). Furthermore, *E. faecalis* can acquire resistance to clinically relevant antibiotics such as vancomycin, linezolid, and kanamycin, complicating treatment options (Govindarajan et al., 2022). Recent studies have highlighted a troubling increase in linezolid-resistant *E. faecalis* strains, with mechanisms of resistance linked to genes such as *erm*(A) and *optr*A, and mutations like G2576U in the 23S rRNA (Ma et al., 2021; Yang et al., 2024). This trend is corroborated by research in China, which reported a 22.61% prevalence of linezolid-resistant *E. faecalis* isolates, primarily associated with the presence of the *erm*(A) gene and risk factors such as indwelling catheters (Ma et al., 2021). Similarly, a study in India identified high rates of *optr*A gene-mediated resistance among *E. faecium* strains, illustrating the widespread nature of this issue (Rani et al., 2023).

The ability of *E. faecalis* to form biofilms, particularly in catheter-associated urinary tract infections (CAUTIs), further exacerbates its antibiotic resistance (Govindarajan and Kandaswamy, 2022). Biofilm formation is a key virulence mechanism, allowing the bacteria to evade host immune responses and enhancing their survival in harsh conditions (Rahimzadeh et al., 2023; Șchiopu et al., 2023). Recent research highlights the crucial role of virulence factors in *E. faecalis* infections. These include secreted factors like cytolysin (*cyl*A), gelatinase (*gel*E), and hyaluronidase (*hyl*), as well as cell surface proteins such as aggregation substances (*asa*1), enterococcal surface protein (*esp*), endocarditis antigen (*efa*A), and collagen-binding protein (*ace*) (Aung et al., 2023; Comerlato et al., 2013; Zhang et al., 2017). A critical enzyme involved in anchoring many of these surface proteins is sortase. Sortase plays a pivotal role in the assembly of pili, which are essential for bacterial adhesion and biofilm formation. By recognizing a cell-wall sorting (CWS) motif, sortase cleaves and anchors surface proteins to the cell wall, contributing to bacterial virulence (Sivaramalingam et al., 2024). Sortase and its associated pili assembly are attractive targets for antimicrobial interventions due to their vital role in infection and biofilm development (Sivaramalingam et al., 2024).

Moreover, biofilms formed by *E. faecalis* often involve interactions with other species, such as *E. coli*. This dual-species biofilm formation enhances virulence and antibiotic resistance, driven in part by mechanisms like iron metabolism. *E. faecalis* biofilms have been shown to increase iron uptake via ferrous iron transporter proteins, which promotes the survival of both *E. faecalis* and *E. coli* under iron-supplemented conditions, enhancing biofilm resilience and antibiotic resistance (Govindarajan and Kandaswamy, 2022). This symbiotic relationship complicates treatment, as biofilms provide a protective environment that shields bacteria from both the immune system and antibiotics (Govindarajan and Kandaswamy, 2022).

Considering the growing clinical significance of *E. faecalis*, the aim of this study is to explore key attributes, including antibiotic resistance, virulence factors, biofilm formation capacity, and molecular typing through multilocus sequence typing (MLST). Understanding these factors will contribute to the development of more effective therapeutic strategies for combating *E. faecalis* infections.

## 2 Materials and methods

### 2.1 Bacterial isolates

Between March 2021 and April 2022, a total of 300 non-duplicated urine samples were collected from inpatients at Shariati Hospital, Tehran, Iran. These patients were suspected of having a UTI based on clinical symptoms evaluated by healthcare professionals. The cohort included 180 males and 120 females. Inclusion criteria required that patients had not taken antibiotics within 48 h prior to sample collection, exhibited bacterial counts of ≥10^5^ colony-forming units (CFU), and demonstrated at least one UTI symptom, such as fever, increased urinary frequency, painful urination, lower abdominal tenderness, bladder congestion, or hematuria (Shahbazi et al., 2018; Shivaee and Mirshekar, 2019; Komala and Kumar, 2013).

Midstream urine samples were collected aseptically in sterile containers and promptly transported to the clinical microbiology laboratory for examination and culture analysis. Urine samples were inoculated onto blood agar plates using calibrated loops and incubated at 37°C for 24 h. Colony morphology and phenotypic characteristics were visually assessed, and *E. faecalis* identification was performed using standard biochemical tests, including Gram staining, catalase testing, bile esculin hydrolysis, growth in 6.5% sodium chloride, and arabinose fermentation. Confirmation of *E. faecalis* isolates was achieved using a polymerase chain reaction (PCR) assay with specific primers (Table 1) (Shahroodian et al., 2022). All confirmed isolates were preserved in brain-heart infusion (BHI) broth (Merck, England) supplemented with 20% glycerol and stored at −80°C for long-term preservation.

**Table 1:** Oligonucleotide primers used in this study.

Genes	Primer sequence (5′-3′)	Amplicon size (bp)	References
*16srRNA-F*	ATCAAGTACAGTTAGTCTTTATTAG	940	Jahansepas et al. (2018)
*16srRNA-R*	ACGATTCAAAGCTAACTGAATCCAGT		
*esp-F*	AGATTTCATCTTTGATTCTTGG	510	Armin et al. (2017)
*espA-R*	AATTGATTCTTAGCATCTGG		
*efba-F*	GCACAAGTCCCAAAAGGAGC	510	Lee et al. (2021)
*efbaA-R*	AAGTGCGGCTTCAGTAAGGG		
*Asa1-F*	TAGGAGTTGTAGGATTAGCTAC	677	Lee et al. (2021)
*Asa1A-R*	TGTTGTATTCMGCSACTTC		
*ace-F*	AAAGTAGAATTAGATCCACAC	320	Bai et al. (2018)
*aceA-R*	TCTATCACATTCGGTTGCG		
*cylA-F*	ACTCGGGGATTGATAGGC	688	Ma et al. (2021)
*cylA-R*	GCTGCTAAAGCTGCGCTT		
*gelE-F*	TATGACAATGCTTTTTGGGAT	213	Ma et al. (2021)
*gelE-R*	AGATGCACCCGAAATAATATA		
*gdh-F*	GGCGCACTAAAAGATATGGT	530	Bai et al. (2018)
*gdh-R*	CCAAGATTGGGCAACTTCGTCCCA		
*gyd-F*	CAAACTGCTTAG CTCCAATGGC	395	Bai et al. (2018)
*gyd-R*	CATTTCGTTGTCATACCAAGC		
*pstS-F*	CGGAACAGGACTTTCGC	583	Bai et al. (2018)
*pstS-R*	ATTTACATCACGTTCTACTTGC		
*gki-F*	GATTTTGTGGGAATTGGTATGG	438	Bai et al. (2018)
*gki-R*	ACCATTAAAGCAAAATGATCGC		
*aroE-F*	TGGAAAACTTTACGGAGACAGC	459	Bai et al. (2018)
*aroE-R*	GTCCTG TCCATTGTTCAAAAGC		
*xpt-F*	AAAATGATGGCCGTGTATTAGG	456	Bai et al. (2018)
*xpt-R*	AACGTCACCGTTCCTTCACTTA		
*yqiL-F*	CAGCTTAAGTCAAG TAAGTGCCG	436	Bai et al. (2018)
*yqiL-R*	GAATATCCCTTCTGCTTGTGCT		

### 2.2 Antibiotic susceptibility testing

Susceptibility of the isolates to a panel of antibiotics was assessed using the Kirby-Bauer disk diffusion test (Traub et al., 1998). The antibiotics tested included vancomycin (30 μg), penicillin G (10 μg), ampicillin (10 μg), tetracycline (30 μg), minocycline (30 μg), ciprofloxacin (5 μg), levofloxacin (5 μg), gatifloxacin (5 μg), gentamicin (120 μg), nitrofurantoin (300 μg), and linezolid (30 μg), all sourced from Mast Group Ltd., United Kingdom. Minocycline was included because, according to CLSI 2022 and 2023 guidelines, although organisms susceptible to tetracycline are typically susceptible to minocycline, some strains that exhibit intermediate or resistant profiles to tetracycline may still be susceptible to minocycline (Lewis and James, 2022). The results were interpreted in accordance with Clinical and Laboratory Standards Institute guidelines (Lewis and James, 2022), and *E. faecalis* ATCC 29212 served as a reference strain for comparative analysis (Khalil et al., 2022).

### 2.3 Virulence gene identification

Bacterial DNA was extracted using the High Pure PCR Template Preparation Kit (Roche, Germany). The extracted DNA was used as the template for PCR amplification. The presence of virulence factors in *E. faecalis* isolates was examined, targeting key genes such as enterococcal surface protein (*esp*), secreted factors like cytolysin (*cyl*), aggregation substances (*asa*1), endocarditis antigen (*efa*A), collagen-binding protein (*ace*), and gelatinase (*gel*E) genes. The PCR conditions followed established protocols (Karimi et al., 2018), and amplification products were sequenced using an ABI 3730X capillary sequencer (Macrogen, Korea). *E. faecalis* ATCC 29212 served as the reference strain.

### 2.4 Biofilm formation assay

Biofilm formation was quantitatively assessed using the microtiter plate method as outlined in previous studies (Bostanghadiri et al., 2019). Bacterial isolates were cultured in LB broth (Merck) and incubated overnight at 37°C. The cultures were subsequently diluted 1:40 in fresh TSB, and 200 μL of the diluted solution was transferred to the wells of a flat-bottomed polystyrene microtiter plate. The plate was then incubated at 37°C for 48 h. Wells containing only TSB served as negative controls. Following incubation, the plates were gently washed three times with phosphate-buffered saline (PBS; pH 7.2) to remove non-adherent cells. The wells were then fixed with 200 μL of methanol (99.8%, Sigma-Aldrich) for 15 min and allowed to air dry at room temperature. Subsequently, the biofilms were stained with 200 μL of crystal violet (1%, Sigma-Aldrich). Excess dye was removed by washing the wells three times with PBS. The crystal violet bound to the adherent cells was solubilized with 200 μL of acetic acid (33%, Sigma-Aldrich) per well. The amount of biofilm formation was determined by measuring the absorbance at 490 nm (OD 490) using an ELISA reader. Isolates were categorized based on the following criteria: strong-biofilm producers (OD > 4 × OD control), moderate-biofilm producers (2 × OD control < OD ≤ 4 × OD control), weak-biofilm producers (OD control < OD ≤ 2 × OD control), and non-biofilm producers (OD ≤ OD control) (Bostanghadiri et al., 2019). *E. faecalis* ATCC 29212 was used as a negative control, and all biofilm assays were performed in triplicate.

### 2.5 Multi-locus sequence typing

MLST of *E. faecalis* isolates followed established methodology (Zheng et al., 2018). Internal regions of seven housekeeping genes— *gyd* (glyceraldehyde-3-phosphate dehydrogenase), *gdh* (glucose-6-phosphate dehydrogenase), *pst*S (phosphate ATP binding cassette transporter), *yqi*L (acetyl-coenzyme A acetyltransferase), *xpt* (shikimate 5-dehydrogenase)*, gki* (putative glucokinase), and, *aro*E (shikimate 5-dehydrogenase)—were amplified using PCR. Primer sequences and references are detailed in Table 1. PCR reactions included 12.5 μL of 2X PCR Master Mix (Ampliqon, Denmark), 1 μL of each forward and reverse primer, 1 μL of DNA, and 9.5 μL of distilled water. The PCR program consisted of initial denaturation at 95°C for 3 min, followed by 30 cycles of denaturation at 95°C for 30 s, annealing at 52°C for 30 s, and extension at 72°C for 60 s, with a final extension at 72°C for 10 min. Sequences were assigned unique allele numbers based on the *E. faecalis* MLST database, and the allelic profile for each isolate was generated by merging the allelic sequences from the seven genes (Zheng et al., 2018).

### 2.6 Statistical analysis

Statistical analysis was performed using SPSS version 21.0 (SPSS Inc., Chicago, IL, United States). Fisher’s exact test and chi-squared test (χ^2^) were used to evaluate the correlation between biofilm formation, antibiotic resistance, and virulence gene distribution. A *p*-value of <0.05 was considered statistically significant.

## 3 Results

### 3.1 Patient demographics and bacterial isolates

In a cohort of 300 individuals diagnosed with urinary tract infections (UTIs), urine samples were analyzed, and microbiological testing identified *E. faecalis* as the causative agent in 160 cases. Consequently, the prevalence of *E. faecalis* in the studied UTI cases was determined to be 53%. Among these 160 *E. faecalis* isolates, 81 were obtained from male patients and 79 from female patients, yielding a male-to-female ratio of 1.02. The majority of the patients, specifically 45 out of 160, were within the age range of 59 to 68 years.

### 3.2 Antibiotic susceptibility pattern of isolates

**Figure 1:**
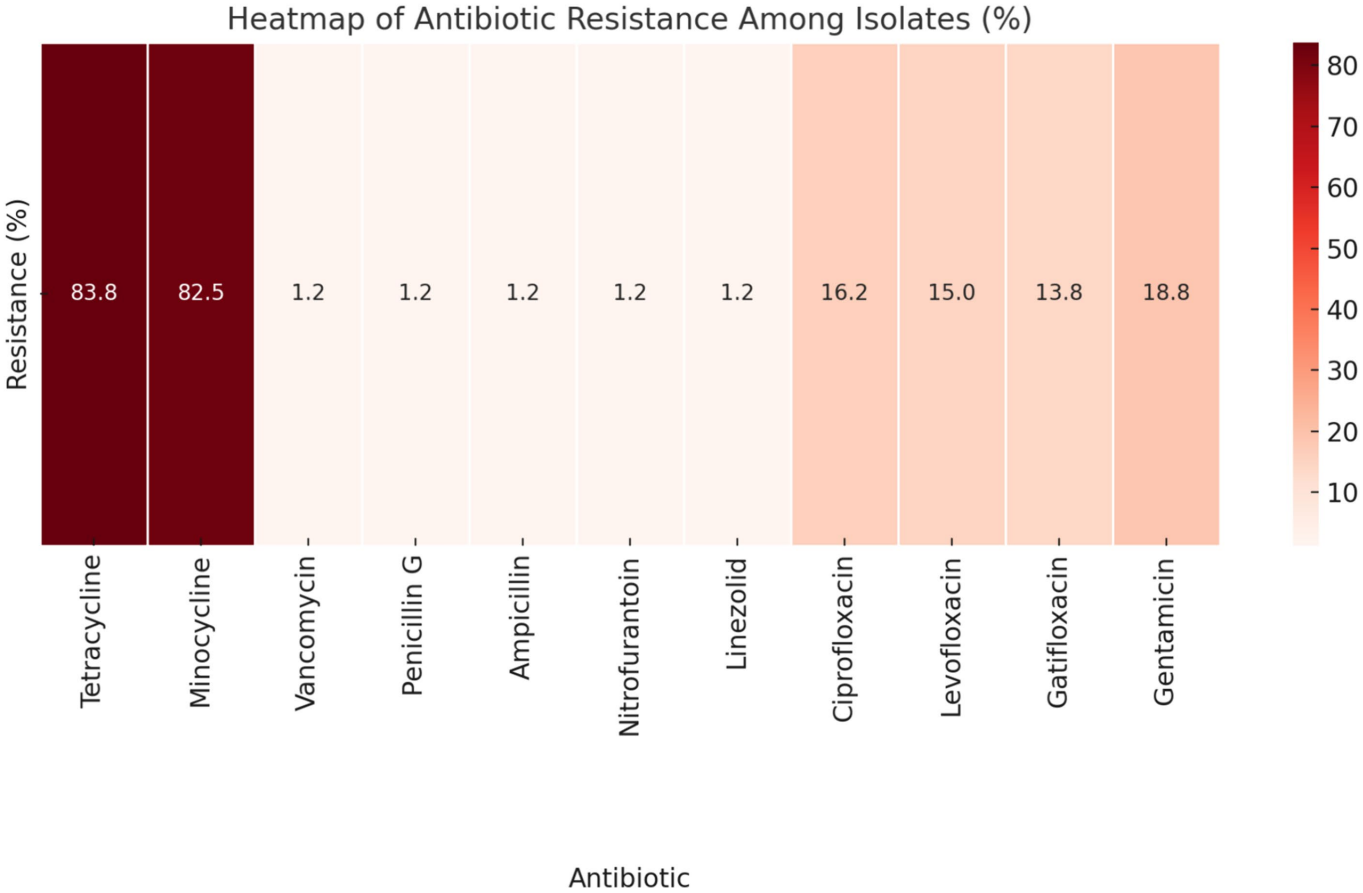
Heatmap displaying the antibiotic resistance patterns among the isolates, according to CLSI interpretation criteria. The darker shades of red indicate higher percentages of resistance, providing a visual representation of how different antibiotics compare in terms of resistance prevalence.

According to the CLSI interpretation criteria, a substantial proportion of the isolates exhibited resistance to tetracycline (83.8%, 134/160) and minocycline (82.5%, 132/160). Conversely, resistance to vancomycin, penicillin G, ampicillin, nitrofurantoin, and linezolid was observed at notably low levels, with prevalence rates of 1.2% (2/160). Resistance to fluoroquinolones was recorded at 16.2% (26/160) for ciprofloxacin, 15.0% (24/160) for levofloxacin, and 13.8% (22/160) for gatifloxacin. Additionally, high-level gentamicin resistance was noted in 18.8% (30/160) of the isolates (Figure 1).

### 3.3 Presence of virulence gene

The presence of the *efa*A and *ace* genes was detected in 93.8% (150/160) of the isolates. For other virulence genes, the distribution was as follows: 72.5% (116/160) of isolates were positive for the *esp* gene, 61.2% (98/160) for the *asa*1 gene, 52.5% (84/160) for the *cyl*A gene, and 88.8% (142/160) for the *gel*E gene.

### 3.4 Biofilm characteristics: phenotypes

Biofilm formation was observed in 25% (40/160) of the isolates. Among these, 15% (24/160) exhibited weak biofilm formation, 8.8% (14/160) displayed moderate biofilm formation, and 1.2% (2/160) demonstrated strong biofilm formation.

### 3.5 Correlation between biofilm formation and antibiotic resistance

**Figure 2:**
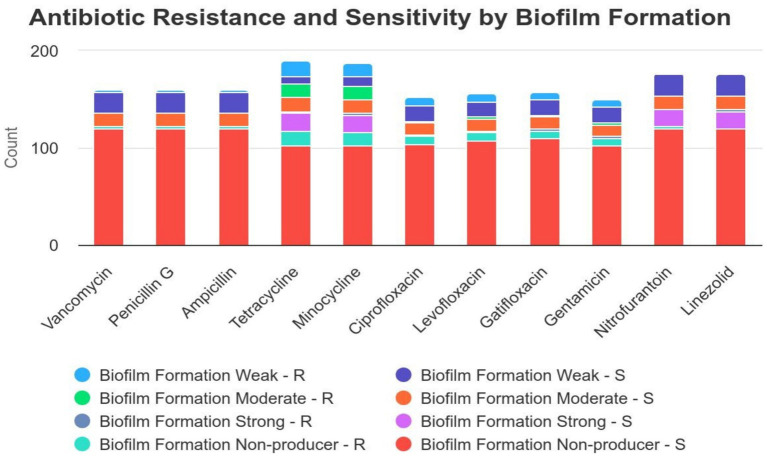
Correlation between biofilm formation and antibiotic resistance distribution in isolates. This figure illustrates the antibiotic resistance and sensitivity patterns among bacterial isolates with different levels of biofilm formation (weak, moderate, strong, and non-producers). The data suggest that isolates with strong biofilm formation exhibit higher resistance to several antibiotics, particularly tetracycline, minocycline, and gentamicin, compared to non-biofilm producers and weak biofilm formers. In contrast, antibiotics such as vancomycin, penicillin G, and nitrofurantoin show low resistance rates across all biofilm formation categories. These findings emphasize the significant role of biofilm formation in increasing bacterial resistance to antibiotics, which is a critical factor to consider when selecting appropriate treatments for infections involving biofilm-forming bacteria.

Although statistical analysis did not reveal a significant correlation between biofilm formation and antibiotic resistance, some noteworthy patterns emerged (Table 2). Among the isolates tested, a subset of vancomycin-resistant (*n* = 2), penicillin G-resistant (*n* = 2), ampicillin-resistant (*n* = 2), and nitrofurantoin-resistant (*n* = 2) isolates exhibited strong biofilm formation. Additionally, tetracycline-resistant isolates demonstrated a range of biofilm production: 16 isolates were classified as weak biofilm producers, 14 as moderate, and 2 as strong biofilm producers. Similarly, minocycline-resistant isolates were categorized as 14 weak, 14 moderate, and 2 strong biofilm producers. For ciprofloxacin, levofloxacin, gatifloxacin, and gentamicin resistance, patterns of biofilm formation included 8 weak, 2 moderate, and 2 strong biofilm producers (Figure 2). Due to the limited number of linezolid-resistant isolates, the relationship between linezolid resistance and biofilm formation remains inconclusive. These observations indicate potential associations that warrant further investigation to establish a definitive link.

**Table 2:** The correlation between biofilm formation and distribution of antibiotic resistance in the isolates.

Antibiotic susceptibility (%)			Biofilm formation (%)			
			Weak	Moderate	Strong	Negative
Vancomycin	R	2 (1.3)	2 (100)	0 (0)	0 (0)	0 (0)
	I	0 (0)	0 (0)	0 (0)	0 (0)	0 (0)
	S	158 (98.8)	22 (13.9)	14 (8.9)	2 (1.3)	120 (75.9)
Penicillin G	R	2 (1.3)	2 (100)	0 (0)	0 (0)	0 (0)
	I	0 (0)	0 (0)	0 (0)	0 (0)	0 (0)
	S	158 (98.8)	22 (13.9)	14 (8.9)	2 (1.3)	120 (75.9)
Ampicillin	R	2 (1.3)	2 (100)	0 (0)	0 (0)	0 (0)
	I	0 (0)	0 (0)	0 (0)	0 (0)	0 (0)
	S	158 (98.8)	22 (13.9)	14 (8.9)	2 (1.3)	120 (75.9)
Tetracycline	R	134 (83.8)	16 (11.9)	14 (10.4)	2 (1.5)	102 (76.1)
	I	0 (0)	0 (0)	0 (0)	0 (0)	0 (0)
	S	26 (16.3)	8 (30.8)	0 (0)	0 (0)	18 (69.2)
Minocycline	R	132 (82.5)	14 (10.6)	14 (10.6)	2 (1.5)	102 (77.3)
	I	0 (0)	0 (0)	0 (0)	0 (0)	0 (0)
	S	28 (17.5)	10 (37.5)	0 (0)	0 (0)	18 (64.3)
Ciprofloxacin	R	26 (16.3)	8 (30.8)	2 (7.7)	2 (7.7)	14 (53.8)
	I	0 (0)	0 (0)	0 (0)	0 (0)	
	S	132 (82.5)	16(12.1)	12 (9.1)	0 (0)	104 (78.8)
Levofloxacin	R	24 (15)	8 (33.3)	2 (8.3)	2 (8.3)	12 (50)
	I	0 (0)	0 (0)	0 (0)	0 (0)	0 (0)
	S	136 (85)	16 (11.8)	12 (8.8)	0 (0)	108 (79.4)
Gatifloxacin	R	22 (13.8)	8 (36.4)	2 (9.1)	2 (9.1)	10 (45.5)
	I	0 (0)	0 (0)	0 (0)	0 (0)	0 (0)
	S	138 (86.3)	16 (11.6)	12 (8.7)	0 (0)	110 (79.7)
Gentamicin	R	30 (18.8)	8 (26.7)	2 (6.7)	2 (6.7)	18 (60)
	I	0 (0)	0 (0)	0 (0)	0 (0)	0 (0)
	S	130 (81.3)	16 (12.3)	12 (9.2)	0 (0)	102 (78.5)
Nitrofurantoin	R	2 (1.3)	2 (100)	0 (0)	0 (0)	0 (0)
	I	0 (0)	0 (0)	0 (0)	0 (0)	0 (0)
	S	158 (98.8)	22 (13.9)	14 (8.9)	2 (1.3)	120 (75.9)
Linezolid	R	2 (1.3)	0 (0)	0 (0)	2 (100)	0 (0)
	I	0 (0)	0 (0)	0 (0)	0 (0)	0 (0)
	S	158 (98.8)	22 (13.9)	14 (8.9)	2 (1.3)	120 (75.9)

S, Sensitive; I, Intermediate; R, Resistant.

### 3.6 The correlation between enterococcal virulence gene distribution and biofilm formation

**Figure 3:**
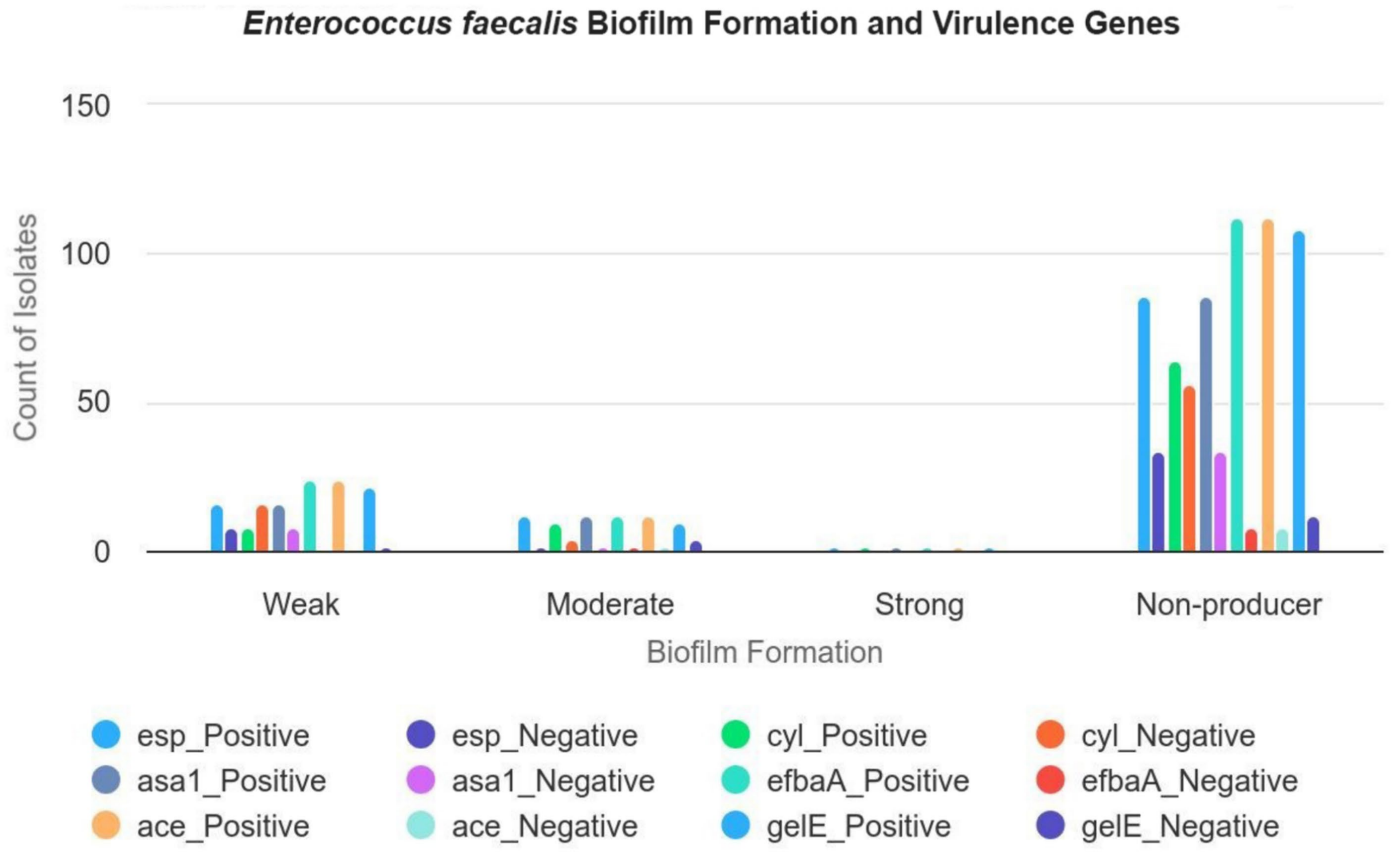
The correlation between the formation of biofilm and the distribution of virulence genes of *E. faecalis* in the isolates. This figure illustrates the relationship between biofilm formation levels (weak, moderate, strong, and negative) and the presence of various virulence genes (*esp., cyl*, *asa1*, *efbaA*, *ace*, and *gelE*) in *E. faecalis* isolates, with a total of 160 isolates. Weak biofilm formers: Out of 24 isolates, 16 carry the *esp* gene, 16 carry the *asa1* gene, and 16 carry the *ace* gene. All 24 isolates exhibit the *efbaA* and *gelE* genes. Moderate biofilm formers: Out of 14 isolates, 12 carry the *esp* gene, 10 carry the *cyl* gene, and 8 carry the *asa1* gene. All 14 isolates carry the *efbaA*, *ace*, and *gelE* genes. Strong biofilm formers: Out of 2 isolates, both carry each of the six virulence genes (*esp., cyl*, *asa1*, *efbaA*, *ace*, and *gelE*). Negative biofilm formers: Out of 120 isolates, 86 carry the *esp* gene, 64 carry the *cyl* gene, and 72 carry the *asa1* gene. 112 carry the *efbaA* and *ace* genes.

This study examined the potential association between biofilm formation and the presence of enterococcal virulence genes, as summarized in Table 3. Although statistical analysis did not reveal a significant correlation between biofilm formation and the presence of these virulence genes, several patterns were observed. Specifically, isolates harboring the *esp* gene (16 isolates), *cyl* gene (8 isolates), *asa*1 gene (16 isolates), *efb*A gene (24 isolates), *ace* gene (24 isolates), and *gel*E gene (22 isolates) were predominantly weak biofilm producers. Conversely, isolates with the *esp* gene (12 isolates), *cyl* gene (10 isolates), *asa*1 gene (8 isolates), *efb*A gene (12 isolates), *ace* gene (12 isolates), and *gel*E gene (10 isolates) displayed moderate biofilm formation. Notably, two isolates that exhibited the full complement of tested virulence genes (*esp., cyl*, *asa*1, *efb*A, *ace*, and *gel*E) were identified as strong biofilm producers (Figure 3). These observations suggest potential associations that warrant further investigation to elucidate the underlying mechanisms.

**Table 3:** The correlation between the formation of biofilm and the distribution of virulence genes of *E. faecalis* in the isolates.

Biofilm formation	*E. faecalis* virulence genes (%)												*P*-Value Pearson Chi-Square
	*esp*		*cyl*		*asa1*		*efba*A		*ace*		*gel*E		
	+	−	+	−	+	−	+	−	+	−	+	−	
Weak	16 (66.7)	8 (33.3)	8 (33.3)	16 (66.7)	16 (66.7)	8 (33.3)	24 (100)	0 (0)	24 (100)	0 (0)	22 (91.7)	2 (8.3)	0.09
Moderate	12 (85.7)	2 (14.3)	10 (71.4)	4 (28.6)	8 (57.1)	6 (42.9)	12 (85.7)	2 (14.3)	12 (85.7)	2 (14.3)	10 (71.4)	4 (28.6)	
Strong	2 (100)	0 (0)	2 (100)	0 (0)	2 (100)	0 (0)	2 (100)	0 (0)	2 (100)	0 (0)	2 (100)	0 (0)	
Negative	86 (71.7)	34 (28.3)	64 (53.3)	56 (46.7)	72 (60)	48 (40)	112 (93.3)	8 (6.7)	112 (93.3)	8 (6.7)	108 (90)	12 (10)	0.3
Total	116 (72.5)	44 (27.5)	84 (52.5)	76 (47.5)	98 (61.2)	62 (38.8)	150 (93.8)	8 (6.7)	150 (93.8)	8 (6.7)	142 (88.8)	18 (11.2)	−

+, Positive; −, Negative.

### 3.7 MLST analysis

The MLST analysis revealed that the two linezolid-resistant *Enterococcus faecalis* isolates, both identified as strong biofilm producers, were of the same sequence type (ST). Specifically, these isolates were classified as ST150, with the following allelic profile: 3, 6, 23, 12, 1, 10, 7.

## 4 Discussion

*E. faecalis* is recognized as a significant Gram-positive pathogen in UTIs, exhibiting notable resistance to a range of commonly used antibiotics such as macrolides and cephalosporins. This resistance arises from both intrinsic factors and acquired mechanisms (Gilmore et al., 2020). Our study highlights a substantial prevalence of antimicrobial resistance among clinical isolates of *E. faecalis* from UTIs, with particularly pronounced resistance observed against minocycline and tetracyclines. Notably, all *E. faecalis* isolates, with the exception of two resistant to linezolid, maintained susceptibility to vancomycin, ampicillin, penicillin G, and nitrofurantoin.

These findings are consistent with the reports by Ma et al. (2021) and Chen et al. (2017), who also noted an increase in resistance to minocycline and tetracyclines while finding that all isolates were susceptible to vancomycin and ampicillin. This observation suggests minimal cross-resistance between linezolid and other antibiotics in the *E. faecalis* isolates studied.

A global perspective on linezolid resistance, as indicated by Dadash et al., reveals that while linezolid resistance is generally low, it exhibits significant regional variability, with higher prevalence observed in Asia compared to other regions (Dadashi et al., 2021). These data corroborate our findings and underscore the importance of localized surveillance in effectively understanding and addressing resistance patterns. The regional variability emphasizes the need for tailored approaches in managing antimicrobial resistance and highlights the value of region-specific data in formulating effective treatment strategies.

Recent research has highlighted the frequent use of antibiotics such as aminoglycosides and nitrofurantoin in the treatment of UTIs caused by vancomycin-resistant *E. faecalis* (Zhanel et al., 2001; Meena et al., 2017; Levitus et al., 2023). This prevalent exposure may exert selective pressure that contributes to the emergence and persistence of resistant *E. faecalis* strains. These observations underscore the critical need for stringent antibiotic stewardship to curb the development of resistance. In our study, we observed a relatively lower rate of resistance to vancomycin and nitrofurantoin compared to findings reported by Tripathi et al. (2016) and Meena et al. (2017). These studies document a troubling increase in resistance to these essential antibiotics, which are crucial for managing nosocomial enterococcal infections. The observed discrepancy in resistance patterns highlights the importance of continuous surveillance and research to adapt treatment strategies effectively and maintain the efficacy of these key antimicrobial agents. The divergence between our findings and those in the literature emphasizes the necessity for vigilant monitoring of resistance trends to vancomycin and nitrofurantoin. Such efforts are vital for ensuring the continued availability of effective therapeutic options for managing severe and complex UTIs (Rahbar et al., 2007).

The widespread use of antimicrobial agents has led to a notable rise in multidrug-resistant (MDR) Gram-positive bacteria, posing significant challenges in clinical settings (Patel et al., 2013). Linezolid, a last-resort antimicrobial for Gram-positive infections, has become a cornerstone in treating such resistant strains (Koulenti et al., 2020). However, the increasing use of linezolid has spurred the emergence of linezolid-resistant strains. Our study identified a linezolid resistance rate of 1.2% (2/160) among *E. faecalis* isolates, which is lower compared to the 3.5 and 3.4% reported by Chen et al. (2018) and Wang et al. (2021) respectively. Moreover, the finding that 1.8% of vancomycin-resistant *E. faecalis* isolates were also resistant to linezolid underscores a critical limitation in treatment options (Cho et al., 2018). Alarmingly, all linezolid-resistant isolates in our study were also vancomycin-resistant, indicating a potential crisis in managing these infections.

The detection of ST150 in our inpatients suggests its potential adaptation to the hospital environment and acquisition of multidrug resistance. The presence of ST150 in a clinical setting raises concerns about its potential as a problematic strain, especially given its broad-spectrum antibiotic resistance. Recent findings indicate that strains from high-risk clonal complexes (CCs), associated with human infections, have also been found in animals (Freitas et al., 2011). This underlines the need for targeted research into ST150’s genetic mechanisms and its impact on clinical outcomes to develop effective interventions and mitigate its dissemination in healthcare settings (Ma et al., 2021).

Biofilm formation by *E. faecalis* in UTIs is a significant concern, especially in the context of catheter use. Our study observed that 15% of isolates showed weak, 8.8% moderate, and 1.2% strong biofilm formation. Notably, the two isolates with strong biofilm-forming abilities were ST150 and resistant to all tested antibiotics. This suggests that strong biofilm formation, coupled with extensive antibiotic resistance, could exacerbate infection management challenges.

The lower prevalence of *E. faecalis* biofilm formation (25%) in our study compared to previous reports (60–90%) in Europe (Sandoe et al., 2003; Arciola et al., 2008; Duprè et al., 2003) could be due to variations in strain sequence types or methodological differences in biofilm assessment. Factors such as strain variability, operational errors in the microtiter plate assay, and lack of standardized biofilm positivity criteria might contribute to these discrepancies.

The pathogenesis of *E. faecalis* in UTIs involves factors beyond antibiotic resistance, such as colonization, tissue destruction, and evasion of host immune responses. In this study, 93.8% of isolates possessed the *efaA* and *ace* genes. Other virulence genes were present at the following rates: *esp* (72.5%), *asa1* (61.2%), *cylA* (52.5%), and *gelE* (88.8%). These findings align with previously reported data: 98, 100, and 92.6% for the *ace* gene in Poland (Łysakowska et al., 2012), and 90, 89.9, and 92.6% for the *gelE* gene in Italy (Creti et al., 2004) and Iran (Kafil et al., 2013).

The high prevalence of the *efbA* gene among our isolates underscores its importance in UTI virulence. EfbA facilitates adherence to extracellular matrix (ECM) proteins, crucial for virulence in ascending UTI models. The *Ace* protein also binds to ECM proteins, aiding in early-stage colonization. *Gelatinase* (*gel*E) plays a role in bacterial dissemination by degrading fibrin (Karimi et al., 2018). The *esp* gene was found in 72.5% of strains, comparable to rates in Iran (77.9%) (Gulhan et al., 2015), Italy (66.7%) (Creti et al., 2004), India (81%) (Singhal et al., 2014), and Japan (72.2%) (Seno et al., 2005), indicating its role as an adhesin. The *asa1* gene was present in 61.2% of isolates, similar to rates reported in Iran (69.6%) (Arbabi et al., 2016) and Italy (51%) (Creti et al., 2004). The *cylA* gene was identified in 52.5% of strains, consistent with findings in Iran (Nasaj et al., 2016), Japan (Seno et al., 2005), and India (Gupta et al., 2014). The predominance of virulence determinants such as efaA, ace, and gelE in our isolates underscores their significant role in *E. faecalis* pathogenicity. The high prevalence of these factors in our study, compared to others, highlights the need for ongoing surveillance and research. The presence of multiple virulence factors in our isolates suggests a complex interplay between resistance and pathogenicity that warrants further investigation.

In summary, our findings underscore the need for continuous monitoring of antimicrobial resistance and virulence factors in *E. faecalis*. The identification of ST150 and its associated resistance profile, coupled with biofilm-forming capabilities, points to a critical area for future research and intervention. Addressing these challenges will be essential for improving clinical outcomes and managing resistant infections effectively.

## 5 Conclusion

This study highlights the emergence of the ST150 clonal lineage of *Enterococcus faecalis* in Tehran, Iran, with a focus on its role in urinary tract infections (UTIs). The data indicate a significant presence of *E. faecalis* in UTIs, with high resistance rates to tetracycline and minocycline, while maintaining high susceptibility to vancomycin, penicillin G, ampicillin, and nitrofurantoin. Notably, a small percentage of isolates demonstrated resistance to linezolid, with these resistant strains belonging to the previously unreported ST150 lineage. The presence of various virulence factors and the ability to form biofilms among these isolates underline their pathogenic potential. Although no definitive correlation between biofilm formation and antibiotic resistance was found, patterns suggest that biofilm production might be associated with resistance. The study underscores the importance of continuous surveillance and molecular characterization of *E. faecalis* to better understand and address emerging resistance patterns and enhance infection control strategies.

## Data Availability

The datasets presented in this study can be found in online repositories. The names of the repository/repositories and accession number(s) can be found in the article/supplementary material.
